# Resveratrol as a Novel Anti-Herpes Simplex Virus Nutraceutical Agent: An Overview

**DOI:** 10.3390/v10090473

**Published:** 2018-09-03

**Authors:** Giuseppe Annunziata, Maria Maisto, Connie Schisano, Roberto Ciampaglia, Viviana Narciso, Gian Carlo Tenore, Ettore Novellino

**Affiliations:** Department of Pharmacy, University of Naples Federico II, Via Domenico Montesano 49, 80131 Naples, Italy; maria.maisto@unina.it (M.M.); connie.schisano@unina.it (C.S.); roberto.ciampaglia@unina.it (R.C.); viviana.narciso@gmail.com (V.N.); giancarlo.tenore@unina.it (G.C.T.); ettore.novellino@unina.it (E.N.)

**Keywords:** herpes simplex virus, HSV-1, HSV-2, resveratrol, polyphenols, nutraceutical

## Abstract

The herpes simplex virus (HSV) is a common human virus affecting many people worldwide. HSV infections manifest with lesions that occur in different parts of the body, including oral, ocular, nasal, and genital skin and mucosa. In rare cases, HSV infections can be serious and lethal. Several anti-HSV drugs have been developed, but the existence of mutant viruses resistant to these drugs led to the individuation of novel antiviral agents. Plant-derived bioactive compounds, and more specifically polyphenols, have been demonstrated to exert marked anti-HSV activity and, among these, resveratrol (RSV) would be considered a good candidate. The purpose of this manuscript is to review the available literature elucidating the efficacy of RSV against HSV and the main demonstrated mechanisms of action.

## 1. Introduction

The herpes simplex virus (HSV) is a common human, double-stranded DNA virus belonging to the Herpesviridae family, which can persist latently for life in the neurons of infected individuals. Periodically, HSV can restart a lytic-replication cycle, entering, thus, a reactivation process that results in recurrent infection, viral shedding, and transmission to new hosts. HSV infection causes skin lesions that are generally localized at the oral, nasal, and ocular level with HSV-1 infection, whereas with HSV-2, infections most commonly occur at genital-skin and mucosa sites. Although generally HSV-induced lesions are benign, in some instances, HSV infections can be particularly serious, leading to significant morbidity and mortality and, in rare case, viral encephalitis, lymphocytic meningitis, or blindness [1].

According to the World Health Organization, about 3.7 billion people under 50 years old and 417 million people aged 15–49 have HSV-1 and -2 infections, respectively. The prevalence of HSV-2 infections is highest in underdeveloped countries, including Africa and some regions of the Americas. In addition, females are more susceptible to HSV-2 infections than males. Furthermore, the existence of a strong relationship between HSV and the human immunodeficiency virus (HIV) was established that causes a higher susceptibility to HIV infection in individuals infected with HSV-2, and more severe complications of HSV-2 infection in HIV-positive people [2].

Over the years, several pharmacological treatments have been licensed for the treatment of HSV infections. These treatments consist of the use of nucleoside analogues, including acyclovir and its derivatives (penciclovir, famciclovir, and valacyclovir), which act by inhibiting the viral DNA polymerase, thus preventing viral DNA synthesis. In general, these compounds require an initial activation by phosphorilations dependent on viral and host kinases, and may cause the termination of the chain elongation. However, further antiviral agents have been developed, including foscarnet and cidofovir, as second-line therapies against drug-resistant HSV. Unlike first-generation anti-HSV agents, foscarnet does not require any initial phopshorilation, but directly acts by binding to and blocking the pyrophosphate-binding site of the viral DNA polymerase, resulting in impeding normal viral replication. On the other side, cidofovir is a monophosphate analog that requires a phopshorilation independent of viral enzymes. The activated cidofovir-diphosphate is incorporated into the nascent viral DNA strand, but it does not necessarily result in chain termination [3,4].

The existence of drug resistance in HSV guided the research towards the development of novel treatments for HSV infections. In this context, natural products, and more specifically polyphenols, have been reported as promising antiviral agents; moreover, the proven efficacy and almost total absence of side effects contributed to the growing interest in the study of the activities of these compounds [5].

In 1976, one of the first studies demonstrating the antiviral activity of polyphenols was conducted. In particular, it was demonstrated that grape-juice phenolic components, separated through a membrane filtration, exerted an inhibitory activity against poliovirus. The same results were also obtained with grape juice and red wine. Interestingly, it was observed that grape pulp did not exert any antiviral activity, suggesting the role of the polyphenols, which is well-known to be found mostly under the skin [6].

The aim of this paper is to analyze current scientific evidence in order to evaluate the anti-HSV effect of resveratrol (RSV). A literature search was conducted in official scientific databases, including PubMed (http://www.ncbi.nlm.nih.gov/pubmed) and ScienceDirect (http://www.sciencedirect.com). Articles in English were identified using specific keywords (“Resveratrol”; “HSV”; “Herpes simplex virus”; “virus”; “polyphenols”; “antiviral activity”) and combinations of these. After the exclusion of studies not meeting the aim of our manuscript, articles were analyzed and subdivided in in vitro and in vivo studies.

## 2. Resveratrol: From Chemistry to Biology

Firstly described in 1939 by Takaoka as a bioactive component in the *Veratrum grandiflorum* roots [7,8,9], over the years RSV has gained a great interest by scientific research, which has extensively studied its biological activity.

**Figure 1:**
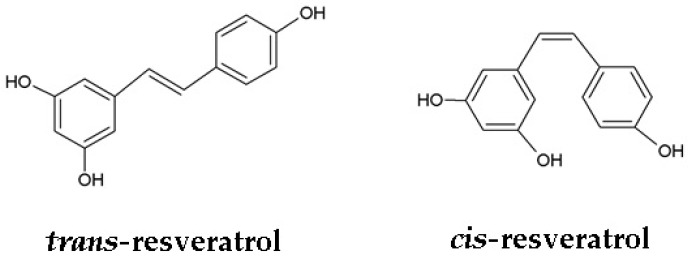
Chemical structure of resveratrol (3,5,4′-trihydroxystilbene) isomers.

Chemically, RSV (3,5,4′-trihydroxystilbene) is a 14-carbon skeleton stilbene with molecular weight 228.25 g/mol. Its structure consists of two aromatic rings with hydroxyl groups in position 3, 5, and 4′, joined by a double styrene bond that is responsible for the existence of the *cis*- and *trans*-RSV isomers (Figure 1) [5]. *Trans*-isomer is known to be more stable than *cis* [10,11].

Stilbenes have been identified in several plant-derived matrices, including grape vines, berries, pines, pomegranates, peanuts, legumes, and soybeans [5,11,12], where they are mainly produced in response to several stimuli, including UV radiation, pathogens, and ozone [11,13,14].

Among the stilbenes, RSV is the main bioactive compound in red wine, and the beneficial effects of the regular consumption of this beverage have been attributed to this compound [15]. However, beside the well-known antioxidant and cardioprotective activities of RSV, further properties of this compound have been described, including neuroprotective [16], phytoestrogenic, and anticancer [11,17] activities. Interestingly, it was demonstrated that RSV is able to induce apoptosis [18,19,20] and cell arrest [21] in cancer cells, suggesting its role in affecting the cell cycle. In addition, not recently the efficacy of RSV as an antibacterial and antiviral agent has been elucidated [5].

As reported in a recent review, much evidence demonstrated the effect of RSV against a number of viruses, including the varicella zoster virus, hepatitis C virus, influenza virus, respiratory syncytial virus, human metapneumonia virus, Epstein–Barr virus, enterovirus, African swine fever virus, duck enteritis virus, and HIV, acting through different mechanisms of action [5]. In addition, Berardi and colleagues clearly demonstrated that in vitro RSV exerted dose-dependent antiviral activity against polyomavirus, affecting the viral progeny DNA synthesis [22]. Furthermore, it was recently demonstrated that RSV at a low μM dose effectively blocked HIV-1 infection in CD4 T cells via a reduction in the levels of deoxynucleoside triphosphate, which are necessary for the reverse transcription of viral RNA; this effect is mainly due to the inhibition of ribonucleotide reductase activity by RSV [23].

## 3. The Role of Resveratrol in HSV Infections

### 3.1. In Vivo Studies

In vivo studies have only been conducted on animal models, mainly on mice. Animals were infected with both HSV-1 and -2, and the effect of RSV on lesion development and/or healing was evaluated. In vivo studies are summarized in Table 1.

In 2004, Dorcherty and colleagues investigated the effects of a cream containing RSV at concentrations of 12.5% and 25% on skin lesions in HSV-1-infected SKH1 mice. Firstly, the authors investigated the effects of RSV in relation to the number of applications and RSV concentration. It was noted that 12.5% RSV cream was not effective in reducing lesion formation when applied two or three times daily, whereas in 25% RSV-treated mice, a significant reduction in lesion formation was observed. On the other hand, when the cream was applied five times daily, both 12.5% and 25% RSV were effective in reducing lesion formation compared to the control. Additionally, it was observed that both 12.5% and 25% RSV were efficacious in limiting lesion formation when applied either 1 or 6 h after infection, but when treatment was started 12 h after infection, improvements were only observed in 25% RSV-treated mice. Furthermore, in a comparison study it was shown that RSV exerted a lesion-suppression effect comparable to that of acyclovir and superior to docosanol. In particular, both 12.5% and 25% RSV, and 5% acyclovir significantly inhibited the development of skin lesions compared to control (*p* = 0.0001). On the other hand, in animals treated with 10% docosanol or control, the observed skin lesions were comparable. Overall, these data suggest that in vivo RSV is effective in reducing skin lesions induced by HSV-1, and its effectiveness depends on RSV concentration, start of treatment time, and number of applications per day [24].

The same research group also carried out an in vivo study aimed to investigate the effect of RSV in reducing vaginal and extravaginal (presence of redness, swelling, papules, ulcers, or eschars in extravaginal tissues)-lesion formation in mice infected with both HSV-1 and -2 [25]. In particular, a 19% RSV cream was tested in comparison with 5% acyclovir ointment (as an active control) and a placebo. All treatments were started 1 h after the virus infection and applied five times daily for 5 days. The authors observed that intravaginal administration of RSV significantly inhibited virus replication on days 1, 3, and 5 (P = 0.042, 0.002, and 0.003, respectively) compared to the placebo; in addition, RSV exerted an efficacy comparable to that of acyclovir. Moreover, 19% RSV significantly limited vaginal HSV-1 infection on days 1, 3, and 5 (*p* = 0.011, 0.014, and 0.007, respectively). Interestingly, in RSV-treated mice, no extravaginal lesions were observed within 11 days after infection, whereas in acyclovir-treated mice extravaginal signs began nine days after. In addition, in acyclovir- and placebo-treated mice, mortality was collectively 10% and 37%, respectively, whereas 3% of RSV-treated mice died [25]. 

**Figure 2:**
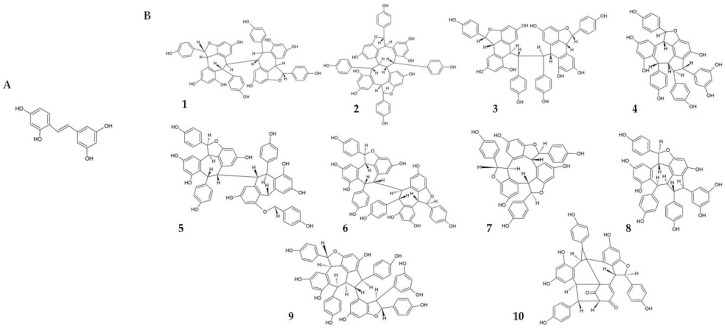
Chemical structures of resveratrol derivatives. (**A**) Oxyresveratrol (trans-2,4,3′,5′-tetrahydroxystilbene); (**B**) oligomeric stilbenoids tested in the study of Chen et al., 2012 [30]: 1, hopeaphenol A; 2, vaticaffinol; 3, davidol A; 4, vaticanol E; 5, neoisohopeaphenol A; 6, pauciflorol C; 7, α-viniferin; 8, pauciflorol B; 9, hemsleyanol D; 10, vaticahainol D.

Among the RSV derivatives, oxyresveratrol (*trans*-2,4,3′,5′-tetrahydroxystilbene) (Figure 2A) has also been demonstrated to exert anti-HSV activity in animal models [26]. Both oral administration and topical application of oxyresveratrol were tested on HSV-1-infected mice. It was observed that 125 mg/kg of oxyresveratrol administered three times daily for seven days, starting 8 h before the virus infection, significantly delayed the development of the lesions (*p* < 0.05) compared with the control; at the dose of 500 mg/kg, a significant delay in development and progression of the lesions (*p* = 0.04) compared with control was observed during 5–8 days treatment. For the topical application, 15% or 30% oxyresveratrol ointment was prepared and applied five times daily for seven days, observing marked dose- and frequency-dependent antiviral activity. In particular, 15% and 30% oxyresveratrol ointment significantly reduced lesion development (*p* = 0.03 and <0.0001, respectively) compared with control. Concerning daily application frequency, 30% oxyresveratrol ointment significantly reduced lesion development when applied five, four, and three times daily (*p* < 0.0001, <0.0001, and <0.004, respectively, compared with control). Moreover, it was shown that 30% oxyresveratrol ointment exerted an efficacy comparable to that of 5% acyclovir cream [26]. 

### 3.2. In Vitro Studies

In vitro studies (Table 1) were also carried out confirming the effects of RSV against HSV observed in vivo and elucidating the main mechanisms of action by which RSV acts.

One of the first in vitro studies investigating the effects of RSV in HSV infections dates back to 1999, when Docherty and colleagues elucidated the main mechanisms of action in monkey-kidney (Vero) and in human-lung cell lines (MRC-5), infected with HSV-1 and HSV-2 [27]. The authors firstly demonstrated that RSV inhibited both HSV-1 and HSV-2 replication in a dose- and time-dependent manner. In particular, at the dose of 25 μg RSV/mL, about 95% virus-replication inhibition was observed, whereas at 50 μg/mL the replication was almost completely abrogated by 72 h. Interestingly, virus replication appeared inhibited as long as RSV was present, whereas, if RSV was removed at 24 and 48 h, virus replication proceeded. According to further explorations conducted by the same authors, RSV affected virus replication by reducing the expression of the protein ICP-4, a regulatory protein essential for HSV replication. In RSV-treated cells, indeed, Western immunoblots analyses revealed that the amount of this protein was lower than that detected in nontreated cells. The reduction of ICP-4 expression mainly resulted from the ability of RSV to inhibit the viral ribonucleotide reductase and kinase activities, resulting in affecting the normal expression of early proteins needed for virus replication and/or reactivation. This process, in turn, caused the arrest of cells in the S/G2 phase [27].

ICP-4 and -27 are two immediate-early virus proteins that play a pivotal role in HSV replication, but they are also responsible for the activation of the nuclear factor kappa-light-chain-enhancer of activated B cells (NF-κB) [31,32] in hosts. NF-κB, in turn, is able to bind the promoter of immediate-early genes, thus, interacting with the virus genome [33]. A not-recent in vitro study demonstrated the ability of RSV to negatively affect NF-κB activation, resulting in (i) inhibition of HSV replication; (ii) alteration of immediate-early, early-, and late-gene activation; and (iii) virus DNA-synthesis inhibition, acting in a dose-dependent manner [28]. In particular, through experiments were conducted on Vero cells, it was demonstrated that at the dose of 219 μM, RSV reversibly suppressed NF-κB activation in HSV-1, -2, and acyclovir-resistant HSV-1-infected cells. Interestingly, authors observed that RSV suppressed NF-κB activation into the nucleus, suggesting that RSV is not able to prevent NF-κB translocation to the nucleus. In addition, it was observed that RSV reduced the transcription of several virus genes, including immediate-early (ICP-0 and -4, whose mRNA was reduced 2.1- and 3.3-fold, respectively) and early (ICP-8 and viral DNA polymerase, whose mRNA was reduced 3.8- and 3.1-fold, respectively) genes, resulting in a significant inhibition of the DNA synthesis (*p* < 0.001, compared to control), with an efficacy comparable to that of acyclovir, and the consequent inhibition of late-gene (glycoprotein C) activation [28].

Recently, a novel mechanism for the RSV anti-HSV activity was elucidated, involving the ability of RSV to activate the 5′ AMP-activated protein kinase/Sirtuin 1 (AMPK/Sirt1) axis [29]. It was previously demonstrated that, during neuronal infection, HSV-1 is able to modulate the AMPK/Sirt1 axis. In particular, AMPK is downregulated during early infection, whereas afterwards (4 h after infection) the levels of AMPK and the phosphorylation of acetyl-CoA carboxylase (one of its substrates) gradually recover; on the other hand, the levels of Sirt1 increase, suggesting that the virus-induced modulation of the AMPK/Sirt1 axis occurs differentially during the infection [34]. Sirt1, in turn, one of the main AMPK targets, inhibits the NF-κB pathway [35]. This suggests, thus, that the inhibition of the AMPK/Sirt1 axis is essential for virus replication. The anti-HSV-1 activity of RSV as AMPK/Sirt1 axis activator was evaluated in Vero and murine hippocampal neuronal (HT22) cell lines, and treated with different RSV concentrations (100, 50, or 10 μM). Firstly, it was observed that cell survival increased by about 20% with 10 μM RSV, and no further increases were observed with higher concentrations. In addition, a significant reduction in the levels of immediate-early-, early-, and late-gene transcript (*p* < 0.001) and HSV-1 proteins (close to 50%) in RSV-treated infected cells was shown. To confirm these results, infected cells were also treated with an AMPK inhibitor, observing an increase in the levels of viral proteins and late-gene transcript, but not of immediate-early and early genes, confirming what was concluded by Martin and coworkers [34] regarding the bimodal activity of HSV-1 on the AMPK/Sirt1 axis. Furthermore, although reductions in viral titer and viral DNA abundance were found in cells treated with 10 μM RSV and the AMPK inhibitors, the best effect was observed in 100 μM RSV-treated cells [29].

In plants, several oligomeric RSV derivatives (also generically named stilbenoids) are widely distributed, which result from the polymerization of RSV residues. The efficacy of a large number of stilbenoids (Figure 2B) against HSV-1 and -2 was tested on Vero cells, showing that trimeric and tetrameric derivatives exerted marked antiviral activity, measured as inhibition of a viral cytopathic effect. In particular, the antiviral activity of these compounds was found greater against HSV-2 than HSV-1. To elucidate the mechanism(s) of action by which these compounds exerted the antiviral activity, several experiments were conducted, concluding that oligomeric stilbenoids inhibited HSV replication not by blocking NF-κB, as demonstrated for RSV, but by increasing the production of Reactive Oxygen Species (ROS). ROS production started within 30 min after infection, with a peak within 2 h [30]. In host cells, ROS are quickly produced as a defence mechanism during infections caused by HSV [36,37], and other members of the Herpesviridae family, such as the Epstein–Barr virus [38] and cytomegalovirus [39]. During the viral infection, ROS play a pivotal role in the activation of innate immune responses. In particular, ROS are involved in the activation and regulation of several processes related to the innate immune system, including autophagy, gene expression, signal transduction, programmed necrosis, and activation of the inflammasome [40,41,42,43,44,45,46]. In addition, it was demonstrated that ROS are also able to induce S-glutathionylation of tumor necrosis factor receptor-associated factor (TRAF) 3 and 6 [36]. Overall, these data suggest the impact of the intracellular redox status on the immune responses against infectious agents. Although polyphenols are historically recognised for their antioxidant activity, evidence demonstrated that, when in excess, they can act as pro-oxidants, increasing the production of free radicals [47]. This may provide an explanation for the ROS-induced antiviral activity of oligomeric RSV derivatives. Furthermore, in vitro evidence demonstrated that oxyresveratrol was able to inhibit the growth of HSV-1 and -2, and acyclovir-resistant HSV-1. In particular, in Vero cells treated with 50 μg/mL oxyresveratrol for 24 and 48 h after infection, a complete inhibition of HSV-1 and -2 replication was observed; furthermore, 26.1% and 32.8% inhibition of HSV-2 replication were obtained in cells treated with 50 μg/mL oxyresveratrol for 3 and 6 h, respectively. Interestingly, similar susceptibility of acyclovir-resistant HSV-1 to oxyresveratrol was observed, suggesting that this compound exerted a mechanism of action different from that of acyclovir. In addition, the authors demonstrated that 50 μg/mL oxyresveratrol affected both the early and late phase of HSV-1 and -2 replication, and that 30 μg/mL oxyresveratrol significantly inhibited the synthesis of late proteins [26]. This finding was corroborated by previous in vitro evidence demonstrating the efficacy of oxyresveratrol and other phenolics from *Millettia Erythrocalyx* and *Artocarpus Lakoocha* against both HSV-1 and -2 [48].

## 4. Conclusions and Future Perspectives

**Figure 3:**
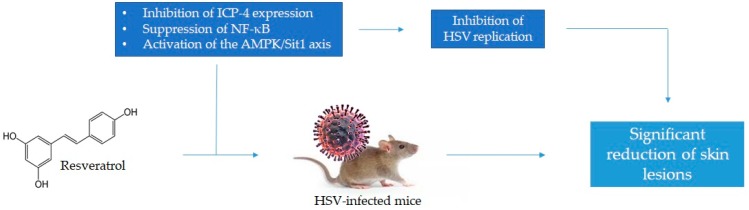
Main mechanisms of action of resveratrol against HSV infection.

In the scientific literature, a limited number of studies demonstrating the effect of RSV against HSV and its role in managing the clinical manifestations are reported. Only three in vivo studies have been reported, and all of these have been conducted on animal models. Additionally, different doses of RSV were used both in vitro and in vivo studies. All evidence, however, is not discordant, and this leads to conclude that RSV is efficacious in limiting or inhibiting virus replication and managing virus infections. In particular, the inhibitory activity of RSV on HSV replication is mainly exerted at the transcriptional apparatus level, where RSV inhibits the ribonucleotide reductase, impairing the normal expression of viral proteins, including ICP-4 that is recognised to be essential for virus replication, and resulting in the alteration of immediate-early, early-, and late-gene activation. This is due to the ability of RSV to act as AMPK/Sirt1 activator and to affect NF-κB activation in the nucleus (Figure 3).

**Figure 4:**
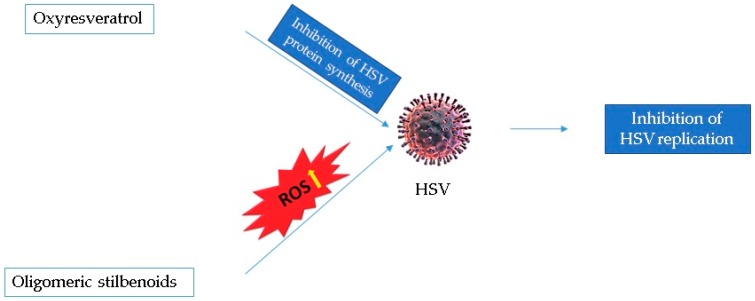
Main mechanisms of action of resveratrol derivatives against HSV infection.

Anti-HSV activity was also demonstrated for RSV derivatives, such as oligomeric stilbenoids that cause an increase in ROS levels, resulting in the activation of the innate immune response, and oxyresveratrol that acts affecting early- and late-gene expression, resulting in the inhibition of virus replication (Figure 4).

Remarkably, the anti-HSV activity of RSV reported in these studies joins the already demonstrated activity against other viruses [5]. Interestingly, for the antiviral activity against different viruses, RSV seems to share similar mechanisms of action, including inhibition of DNA synthesis [21] and ribonucleotide reductase activity [23]. 

As mentioned above, no human studies have been conducted, and this may represent a limitation for the use of RSV in clinical practice as an anti-HSV agent. Studies on humans, thus, are needed in order to confirm the evidence from animal-based and in vitro studies. The high prevalence of labial skin lesions caused by HSV infection in the general population might be a point for randomized clinical trials, controlled with placebo or active controls. The limited cost of raw materials, then, may represent an advantage in this field.

Of note, several food-derived matrices, including agrifood byproducts such as grape pomace, have been reported to contain amounts of RSV and other polyphenols. Based on that, studies aimed to evaluate the anti-HSV activity of RSV-rich food extracts would be interesting. The synergistic effect of RSV and other polyphenols may represent an added value. It has been reported, indeed, that quercetin exerts an anti-HSV activity similar to that of RSV, acting as AMPK activator [28]. However, the activities of RSV and quercetin have been separately monitored; thus, the evaluation of synergistic, or eventually potentiated, activity of these compounds against HSV would be interesting.

Overall, this evidence might drive both scientific research and the pharmaceutical industry towards the development of novel nutraceutical products for the treatment of HSV-induced skin lesions.

## Figures and Tables

**Table 1:** In vivo and in vitro evidences demonstrating the anti-HSV activity of RSV and its derivatives.

Reference	Type of Study	Experimental Model	Treatment	Observed Effect
[24]	In vivo	HSV-1-infected SKH1 mice	12.5% and 25% RSV cream	RSV significantly reduced skin lesions and its effectiveness depended on RSV concentration, start of treatment time, and number of applications per day.
[25]	In vivo	HSV-1- and -2-infected SHK1 mice	19% RSV cream	RSV significantly reduced vaginal lesions with an efficacy comparable to that of acyclovir; in addition, in RSV-treated mice, no extravaginal signs, but significantly reduced mortality were observed.
[26]	In vivo	HSV-1-infected BALB/c Mice	125 mg/kg and 500 mg/kg oxyresveratrol (oral administration)	At the dose of 125 mg/kg, a significant delay in the lesions’ development was observed compared with the control; at the dose of 500 mg/kg, development and progression of the lesions significantly delayed during 5–8 days of treatment.
			15% or 30% oxyresveratrol ointment (topical application)	A marked dose- and frequency-dependent reduction of the mean lesion score was observed.
	In vitro	Vero cells infected with HSV-1 and -2	50 μg/mL oxyresveratrol	Significant inhibition of the HSV-1,-2, and acyclovir-resistant HSV-1 replication
			30 μg/mL oxyresveratrol	Inhibition of HSV-1 protein synthesis
[27]	In vitro	Vero and MRC-5 cell lines infected with HSV-1 and -2	25 and 50 μg/mL RSV	RSV inhibited both HSV-1 and HSV-2 replication in a dose- and time-dependent manner through the inhibition of protein ICP-4 expression.
[28]	In vitro	Vero cells infected with HSV-1, -2, and acyclovir-resistant HSV-1	219 μM RSV	RSV was responsible for the nuclear suppression of the NF-κB activation in infected cells; this suppression is reversible and dose-dependent. In addition, RSV negatively affected the expression of immediate-early, early, and late genes and viral DNA synthesis
[29]	In vitro	Vero and HT22 cell lines infected with HSV-1	10, 50, or 100 μM RSV	RSV significantly reduced immediate-early-, early-, and late-gene transcript and HSV-1 proteins levels by activating the AMPK/Sirt1 axis.
[30]	In vitro	Vero cells infected with HSV-1 and -2	Oligomeric RSV derivatives	Anti-HSV-1 and -2 activity by increasing ROS levels.

HSV, herpes simplex virus; RSV, resveratrol; MRC, human lung cell line; NF-κB, nuclear factor kappa-light-chain-enhancer of activated B cells; HT22, murine hippocampal neuronal cell line; AMPK/Sirt1, 5′ AMP-activated protein kinase/Sirtuin 1; ROS, reactive oxygen species.
